# Inflammatory Pain Reduces C Fiber Activity-Dependent Slowing in a Sex-Dependent Manner, Amplifying Nociceptive Input to the Spinal Cord

**DOI:** 10.1523/JNEUROSCI.3816-16.2017

**Published:** 2017-07-05

**Authors:** Allen C. Dickie, Barry McCormick, Veny Lukito, Kirsten L. Wilson, Carole Torsney

**Affiliations:** Centre for Integrative Physiology, Edinburgh Medical School: Biomedical Sciences, The University of Edinburgh, Edinburgh EH8 9XD, United Kingdom

**Keywords:** conduction velocity slowing, dorsal horn, hyperalgesia, nociceptor, primary afferent, spinal output neurons

## Abstract

C fibers display activity-dependent slowing (ADS), whereby repetitive stimulation (≥1 Hz) results in a progressive slowing of action potential conduction velocity, which manifests as a progressive increase in response latency. However, the impact of ADS on spinal pain processing has not been explored, nor whether ADS is altered in inflammatory pain conditions. To investigate, compound action potentials were made, from dorsal roots isolated from rats with or without complete Freund's adjuvant (CFA) hindpaw inflammation, in response to electrical stimulus trains. CFA inflammation significantly reduced C fiber ADS at 1 and 2 Hz stimulation rates. Whole-cell patch-clamp recordings in the spinal cord slice preparation with attached dorsal roots also demonstrated that CFA inflammation reduced ADS in the monosynaptic C fiber input to lamina I neurokinin 1 receptor-expressing neurons (1–10 Hz stimulus trains) without altering the incidence of synaptic response failures. When analyzed by sex, it was revealed that females display a more pronounced ADS that is reduced by CFA inflammation to a level comparable with males. Cumulative ventral root potentials evoked by long and short dorsal root stimulation lengths, to maximize and minimize the impact of ADS, respectively, demonstrated that reducing ADS facilitates spinal summation, and this was also sex dependent. This finding correlated with the behavioral observation of increased noxious thermal thresholds and enhanced inflammatory thermal hypersensitivity in females. We propose that sex/inflammation-dependent regulation of C fiber ADS can, by controlling the temporal relay of nociceptive inputs, influence the spinal summation of nociceptive signals contributing to sex/inflammation-dependent differences in pain sensitivity.

**SIGNIFICANCE STATEMENT** The intensity of a noxious stimulus is encoded by the frequency of action potentials relayed by nociceptive C fibers to the spinal cord. C fibers conduct successive action potentials at progressively slower speeds, but the impact of this activity-dependent slowing (ADS) is unknown. Here we demonstrate that ADS is more prevalent in females than males and is reduced in an inflammatory pain model in females only. We also demonstrate a progressive delay of C fiber monosynaptic transmission to the spinal cord that is similarly sex and inflammation dependent. Experimentally manipulating ADS strongly influences spinal summation consistent with sex differences in behavioral pain thresholds. This suggests that ADS provides a peripheral mechanism that can regulate spinal nociceptive processing and pain sensation.

## Introduction

The basic currency of communication in the nervous system is the action potential. As the action potential is an all-or-none event, information is coded by the number of action potentials and the time intervals between them. In the 1920s, Edgar Adrian demonstrated that the intensity of a sensation is coded by the firing frequency in afferent nerve fibers in the somatosensory system (Adrian, 1926; Adrian and Zotterman, 1926a,b). Subsequently, microneurography studies in humans have directly demonstrated that the firing frequency in C fiber nociceptors encodes pain intensity (Torebjörk et al., 1984; Yarnitsky and Ochoa, 1990). The more noxious the stimulus, the shorter the intervals between successive action potentials in nociceptive C fibers and the more intense the pain experienced.

Nociceptive C fibers display activity-dependent slowing (ADS), whereby repetitive stimulation results in a progressive slowing of action potential conduction velocity, which manifests as a progressive increase in response latency in both human (Serra et al., 1999; Weidner et al., 1999) and animal (Thalhammer et al., 1994; Gee et al., 1996) studies. Subtypes of C fibers display this phenomenon to differing degrees such that ADS can be used to functionally classify C fiber subtypes (Thalhammer et al., 1994; Serra et al., 1999; Weidner et al., 1999). ADS occurs in a frequency- and length-dependent manner, with greater ADS observed at higher frequencies (Thalhammer et al., 1994; Gee et al., 1996; Serra et al., 1999; Weidner et al., 1999) and over longer lengths (Schmelz et al., 1995; Zhu et al., 2009). This progressive slowing of conduction velocity presumably regulates the intervals between successive action potentials reaching the spinal cord, which could influence central pain processing and pain sensation; however, this has not been investigated.

ADS involves voltage-gated sodium (Nav) channels (De Col et al., 2008; Obreja et al., 2012), likely Nav1.7 and Nav1.8 (Baker and Waxman, 2012; Petersson et al., 2014; Tigerholm et al., 2014; Hoffmann et al., 2016), and is constrained by hyperpolarization-activated cyclic nucleotide-gated (HCN) channels (Takigawa et al., 1998; Zhu et al., 2009; Mazo et al., 2013). These channels are known to be regulated in inflammatory pain (Emery et al., 2012; Weng et al., 2012; Rahman and Dickenson, 2013; Waxman and Zamponi, 2014), suggesting that C fiber ADS may be altered in inflammatory pain. Specifically, CFA inflammation increases both hyperpolarization-activated current (*I*_h_) and HCN2 expression levels in C fiber nociceptors (Papp et al., 2010; Acosta et al., 2012; Weng et al., 2012) and alters *I*_h_ activation properties (Djouhri et al., 2015). Furthermore, deletion of HCN2 in Nav1.8-expressing neurons, which are mainly C fiber nociceptors, limits inflammatory thermal hyperalgesia (Emery et al., 2011), and peripheral block of HCN channels attenuates inflammatory pain (Young et al., 2014). There is also a loss of inflammatory pain phenotype in Nav1.7 and Nav1.8 knock-out mice and in mice in which Nav1.7 was selectively deleted in Nav1.8-expressing neurons (Akopian et al., 1999; Nassar et al., 2004, 2005). Furthermore, Nav1.7 and Nav1.8 channel expression is increased in CFA inflammation (Coggeshall et al., 2004; Gould et al., 2004; Liang et al., 2013), and selective blockers of either channel reduce inflammatory pain (McGowan et al., 2009; Zhang et al., 2010; Bregman et al., 2011; Yang et al., 2013; Lee et al., 2014; Payne et al., 2015).

The aim of this study was, therefore, to determine whether C fiber ADS is altered in the CFA inflammation model and the impact on temporal relay of nociceptive input to the spinal cord. Given increasing awareness of sex differences in pain sensitivity and injury-induced hypersensitivity (Mogil and Bailey, 2010; Mogil, 2012; Bartley and Fillingim, 2013), this was also investigated in both sexes. Furthermore, the impact of ADS on spinal summation and output was explored using electrophysiological and behavioral analysis of the nociceptive flexion withdrawal reflex.

## Materials and Methods

### 

#### 

##### Animals.

All experiments were approved by the University of Edinburgh Ethical Review Committee and performed in accordance with the UK Animals (Scientific Procedures) Act 1986. Sprague Dawley rats of both sexes (University of Edinburgh Biological Research Resources) were used in all experiments. Animals were housed in cages at 21°C and 55% relative humidity, with a 12 h light/dark cycle, and food and water were provided *ad libitum*.

##### Inflammatory pain model.

To induce peripheral inflammation, juvenile rats received an intraplantar injection of complete Freund's adjuvant (CFA; 0.5 mg/ml saline) into the left hindpaw (1 μl/g body weight) under isoflurane anesthesia, at approximately postnatal day 18 (P18), 2–5 d before patch-clamp or compound action potential electrophysiological recording at approximately P21. This procedure results in persistent peripheral hindpaw inflammation and behavioral hypersensitivity in rats of this age (Torsney, 2011). Control rats were untreated.

##### Isolated dorsal root preparation.

Isolated dorsal roots were prepared as described previously (Torsney, 2011; Dickie and Torsney, 2014). Briefly, naive untreated (control) or CFA-treated (approximately P21) rats were decapitated under isoflurane anesthesia, and spinal cords, with attached dorsal roots, were removed in ice-cold dissection solution. Lumbar (L4/L5) dorsal roots (left side only, CFA treated) were cut near the dorsal root entry zone, and their dorsal root ganglia were removed, before being placed in 36–37°C oxygenated recovery solution for 1 h. Roots were transferred to the recording chamber of an upright microscope (Ziess) and perfused with oxygenated Krebs' solution (1–2 ml/min) at room temperature. The 95% O_2_/5% CO_2_-saturated Krebs' solution contained (in mm) 125 NaCl, 2.5 KCl, 1.25 NaH_2_PO_4_, 26 NaHCO_3_, 25 glucose, 1 MgCl_2_, and 2 CaCl_2_, pH 7.4. Recovery solution was identical to Krebs' solution apart from 1.5 mm CaCl_2_ plus 6 mm MgCl_2_. Dissection solution was the same as the recovery solution, but with 1 mm kynurenic acid.

##### Compound action potential recording.

Two glass suction electrodes were used, one for electrical orthodromic stimulation and the second for recording compound action potentials (CAPs). Dorsal roots were stimulated 10 times at 0.2 Hz (0.1 ms duration), with an ISO-flex stimulus isolator (A.M.P.I.), at 1, 2, 3, 4, 5, 7.5, 10, 15, 20, and 25 μA; then in 10 μA steps between 30 and 100 μA and in 50 μA steps between 150 and 500 μA (Torsney, 2011; Dickie and Torsney, 2014). A 0.1 ms pulse width was chosen to replicate the electrical stimuli previously established to activate the different afferent fiber types in this age of rat (Nakatsuka et al., 2000). However, the possibility of an underestimation of the C fiber contribution cannot be excluded given that longer pulse widths have also been used to stimulate C fiber inputs (Baba et al., 1999). The main components of the compound action potentials were differentiated as Aβ, Aδ, and C fiber on the basis of activation threshold and conduction velocity, each displaying a characteristic triphasic (positive–negative–positive) response. Data were acquired and recorded using an ER-1 differential amplifier (Cygnus Technologies) and pClamp 10 software (Molecular Devices). Data were filtered at 10 kHz and sampled at 50 kHz.

The activation threshold was defined as the lowest stimulation intensity at which the negative component of the triphasic response was clearly identifiable. The amplitude of each component was calculated by measuring the distance between the negative and second positive peaks. The conduction velocity was calculated based on the latency to the negative peak at 20, 100, and 500 μA for the Aβ, Aδ, and C components, respectively.

To assess ADS, dorsal roots were stimulated 16 or 40 times (500 μA intensity, 0.1 ms duration) at frequencies of 1 or 2 Hz. For each stimulus, the latency between the stimulus artifact and the negative peak of the triphasic response was measured, and the change in latency from stimulus 1 was calculated. In some cases, the width of the C fiber component (positive peak to positive peak) was additionally measured, and the change in width from stimulus 1 was calculated. To negate any influence of varying dorsal root length, the latency/width change was normalized to the length of root stimulated, measured as the distance between the stimulating and recording electrodes. In a subset of recordings, the stimulating electrode was first placed close to the distal end of the dorsal root (long stimulation length) and then placed closer to the recording electrode (short stimulation length). By subtracting the latency change values for “short stimulation length” from “long stimulation length,” the latency change solely attributable to conduction velocity slowing, independent of action potential initiation, was calculated.

##### Spinal cord slice preparation.

Spinal cords with attached dorsal roots, from which dorsal root ganglia were removed, were obtained from control or CFA-treated (approximately P21) rats, as described above. The lumbar (L4/L5) segment was embedded in an agarose block, and 350 μm slices, with attached dorsal roots (left side only, CFA treated), were cut. Slices were placed in oxygenated recovery solution at 36–37°C for 1 h and incubated at room temperature for 30 min with 35 nm tetramethylrhodamine-conjugated substance P (TMR-SP), as described previously (Labrakakis and MacDermott, 2003; Torsney, 2011; Dickie and Torsney, 2014). Slices were allowed to recover for an additional 1 h at room temperature before being transferred to the recording chamber of an upright microscope (Ziess), equipped with fluorescence for the identification of TMR-SP-labeled (TMR-SP+) neurons and infrared differential interference contrast for electrophysiological recordings, and were continually perfused with oxygenated Krebs' solution (1–2 ml/min) at room temperature.

##### Patch-clamp recording.

Whole-cell patch-clamp recordings (holding potential, −70 mV) were made from TMR-SP+ neurons in the lamina I region of the dorsal horn. The intracellular solution used was composed of (in mm) 120 Cs-methylsulfonate, 10 Na-methylsulfonate, 10 EGTA, 1 CaCl_2_, 10 HEPES, 5 *N*-(2,6-dimethylphenylcarbamoylmethyl)triethylammonium chloride (QX-314-Cl), and 2 Mg^2+^-ATP (pH adjusted to 7.2 with CsOH; osmolarity, 290 mOsm), and junction potential was corrected before recording. Additionally, 1 μm Alexa Fluor 488 hydrazide was included in the recording pipette. Data were recorded and acquired with an Axopatch 200B amplifier and pClamp 10 software (Molecular Devices). Data were filtered at 5 kHz and digitized at 10 kHz.

Monosynaptic primary afferent input to lamina I neurokinin 1 receptor-positive (NK1R+) neurons was identified as described previously (Torsney and MacDermott, 2006; Torsney, 2011; Dickie and Torsney, 2014). Evoked EPSCs (eEPSCs) were recorded in response to low-frequency (0.05 Hz) dorsal root stimulation (three times) at intensities of 20, 100, and 500 μA (0.1 ms stimulus duration) to activate Aβ, Aδ, and C fiber inputs, respectively, using an ISO-flex stimulus isolator. To characterize an input as monosynaptic or polysynaptic, dorsal roots were stimulated (20 times) at the following intensities and frequencies: Aβ, 20 μA/20 Hz; Aδ, 100 μA/2 Hz; C, 500 μA/1 Hz. A fiber responses were considered monosynaptic if they displayed no synaptic failures and a stable latency (≤2 ms), whereas C fiber inputs were considered monosynaptic if they displayed no synaptic failures, regardless of whether there was latency variability (Nakatsuka et al., 2000).

To assess whether monosynaptic Aδ or monosynaptic C fiber input to lamina I NK1R+ neurons displayed ADS in response to repetitive stimulation, eEPSCs were recorded in response to trains of 16 stimuli delivered at 1 or 2 Hz (Aδ and C) or trains of 40 stimuli delivered at 2, 5, or 10 Hz (C only), at intensities of 100 μA (Aδ) or 500 μA (C). The latency of each eEPSC was measured as the time between the stimulus artifact and the onset of the monosynaptic response, and the change in latency from stimulus 1 was calculated. These latency change data were also normalized to dorsal root length, measured as the distance between the stimulating electrode and the dorsal root entry zone, to account for variations in the length of dorsal root stimulated.

##### Dorsal root–ventral root potential recording.

Control rats (approximately P10) were decapitated under isoflurane anesthesia, and spinal cords, with attached dorsal and ventral roots, were removed in ice-cold dissection solution. Hemisected lumbar spinal cord with only L4/L5 dorsal and ventral roots left attached were prepared (Otsuka and Konishi, 1974) and transferred to a recording chamber perfused with oxygenated Krebs' solution (>2 ml/min) at room temperature. Two glass suction electrodes were used, one for electrical stimulation of the dorsal root and the second for recording ventral root potentials. The stimulating electrode was first placed close to the distal end of the dorsal root (long stimulation length/more ADS) and then placed closer to the spinal cord (short stimulation length/less ADS) to assess the impact of length-dependent ADS on spinal summation. The dorsal root was stimulated 40 times at 2, 5, and 10 Hz (500 μA intensity, 0.1 ms duration) at both sites, and the cumulative ventral root potential was recorded using a close-fitting glass electrode placed on the ventral root close to the ventral horn (Thompson et al., 1994). Data were acquired and recorded using an ER-1 differential amplifier (Cygnus Technologies) and pClamp 10 software (Molecular Devices). Data were filtered at 10 kHz and sampled at 50 kHz.

##### Sensory testing.

Before and 2–5 d after intraplantar CFA injection (as detailed above), hindpaw swelling and mechanical and thermal sensitivity were measured. Hindpaw swelling was assessed by measuring the thickness of the dorsoventral paw using Vernier calipers. After habituation on an elevated mesh platform, the mechanical threshold of the nociceptive flexion withdrawal reflex was determined with von Frey filaments (Stoelting) applied to the midplantar surface of the hindpaw using the up–down method (Chaplan et al., 1994). After habituation to the Hargreaves apparatus, radiant heat was applied to the midplantar surface of the hindpaw (three times stimuli per hindpaw to calculate average) to determine the noxious thermal withdrawal latency.

##### Statistical analysis.

Area under the curve (AUC) analysis was used to compare ADS in both CAP and patch-clamp recordings. Group comparisons, for both electrophysiology and behavioral data, were performed using two-way ANOVA with or without repeated-measures analysis as appropriate, followed by Sidak's multiple comparisons test or Tukey's multiple comparisons test if an interaction between factors was observed. Averaged data are represented as mean ± SE.

##### Materials.

All chemicals were obtained from Sigma, except TMR-SP (Enzo Life Sciences), Alexa Fluor 488 hydrazide (Invitrogen), QX-314-Cl (Alomone Labs), and NBQX (Tocris Bioscience).

## Results

### CFA inflammation reduces C fiber ADS in isolated dorsal roots

**Figure 1.**
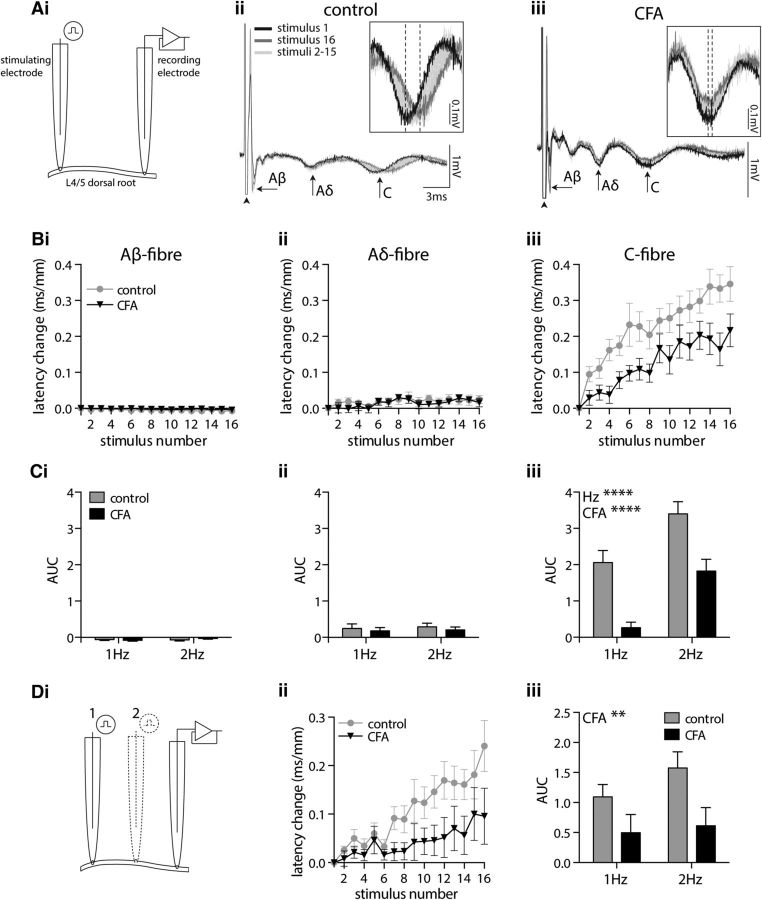
CFA inflammation reduces C fiber ADS in isolated dorsal roots. ***Ai***, Two suction electrodes were used to stimulate and record compound action potentials from L4/L5 dorsal roots. ***Aii***, ***Aiii***, Representative compound action potentials recorded from dorsal roots isolated from control (***Aii***) and CFA-treated (***Aiii***) rats, illustrating the fast (Aβ), medium (Aδ), and slow (C) conducting components. The 16× traces recorded in response to 2 Hz dorsal root stimulation are shown. Arrowheads indicate the stimulus artifact. Insets magnify the C fiber component, with dashed lines indicating the negative peaks of the first and last responses. ***Bi–Biii***, Repetitive stimulation of dorsal roots at 2 Hz results in a negligible change in the latency of Aβ (***Bi***) or Aδ (***Bii***) fiber responses, whereas C fibers display a progressive increase in response latency (***Biii***). ***C***, AUC analysis of latency change reveals that CFA inflammation reduces the frequency-dependent progressive latency change observed in C fibers (2-way ANOVA; CFA, ****p < 0.0001; frequency, ****p < 0.0001). ***Di–Diii***, Eliminating the contribution of action potential initiation time to latency change, by subtracting the latency values obtained at the short (position 2) from the long (position 1) root stimulation length (***Di***), confirms that CFA attenuates C fiber ADS (***Dii***, ***Diii***; 2-way ANOVA; CFA, **p = 0.009). Data in ***B*** and ***C***: Aβ control, *n* = 10; CFA, *n* = 12; Aδ/C control, *n* = 11; CFA, *n* = 13. Data in ***D***: control, *n* = 10; CFA, *n* = 12.

To assess the impact of CFA inflammation on C fiber ADS, dorsal roots isolated from control or CFA-treated rats were repetitively stimulated, and the response latencies of Aβ, Aδ, and C fiber compound action potentials were measured. The Aβ, Aδ, and C fiber afferent components were identified on the basis of conduction velocity and activation threshold (Fig. 1*A*). CFA inflammation did not alter threshold stimulus intensity (*p* = 0.394: Aβ control (*n* = 10), 7.6 ± 0.80 μA; Aβ CFA (*n* = 12), 5.9 ± 0.60 μA; Aδ control (*n* = 11), 38.6 ± 3.0 μA; Aδ CFA (*n* = 13), 34.6 ± 2.8 μA; C control (*n* = 11), 240.9 ± 14.8 μA; C CFA (*n* = 13), 226.9 ± 15.6 μA), conduction velocity (*p* = 0.418: Aβ control, 4.6 ± 0.50 m/s; Aβ CFA, 5.0 ± 0.30 m/s; Aδ control, 0.9 ± 0.1 m/s; Aδ CFA, 0.7 ± 0.1 m/s; C control, 0.2 ± 0.01 m/s; C CFA, 0.2 ± 0.01 m/s), or amplitude (*p* = 0.091: Aβ control, 1.7 ± 0.2 mV; Aβ CFA, 2.4 ± 0.40 mV; Aδ control, 0.1 ± 0.02 mV; Aδ CFA, 0.1 ± 0.02 mV; C control, 0.1 ± 0.02 mV; C CFA, 0.1 ± 0.03 mV), as previous studies have demonstrated in both adult (Baba et al., 1999; Nakatsuka et al., 2000) and similarly aged juvenile (Torsney, 2011) rats.

Repetitive stimulation of isolated dorsal roots produced a negligible reduction (speeding) in the latency of the Aβ fiber response (Fig. 1*Bi*,*Ci*) and a marginal increase (slowing) in the Aδ fiber response latency (Fig. 1*Bii*,*Cii*). CFA inflammation did not alter these observations in A fibers (Aβ, *p* = 0.693; Aδ, *p* = 0.451). In contrast, repetitive stimulation resulted in a clear progressive increase in C fiber response latency (Fig. 1*Biii*) that was confirmed to be frequency dependent, with 2 Hz stimulation resulting in greater ADS than 1 Hz stimulation (*p* < 0.0001; Fig. 1*Ciii*). Notably, this C fiber ADS was significantly reduced by CFA inflammation, independent of stimulation frequency (*p* < 0.0001; Fig. 1*Biii*,*Ciii*).

To address the possibility that the observed CFA effect may reflect altered action potential initiation time rather than altered conduction velocity slowing, compound action potentials were recorded with the stimulating electrode placed at two different positions on an individual dorsal root (Fig. 1*Di*). Subtraction of the C fiber latency values recorded after stimulation at position 2 (short stimulation length) from those stimulated at position 1 (long stimulation length) eliminates the contribution of action potential initiation, leaving only conduction time (between the two stimulation sites). This also revealed a progressive increase in C fiber response latency (Fig. 1*Dii*) that was reduced by CFA inflammation independent of stimulus frequency (*p* = 0.009; Fig. 1*Dii*,*Diii*). This suggests that altered action potential initiation time is not, therefore, likely to account for the observed CFA inflammation-dependent reduction in C fiber ADS. Furthermore, the demonstration that subtracting the short from the long stimulation length results in a progressive increase in the C fiber response latency confirms the length dependency of the phenomenon.

### CFA inflammation reduces ADS in monosynaptic C fiber input to lamina I NK1R+ neurons

To explore the spinal impact of CFA-reduced C fiber ADS, whole-cell patch-clamp recordings were made from lamina I NK1R+ neurons, which are likely projection neurons (Marshall et al., 1996; Todd et al., 2000; Spike et al., 2003; Al-Khater et al., 2008) that receive monosynaptic input from both C and Aδ fibers (Torsney and MacDermott, 2006; Torsney, 2011; Peirs et al., 2015). Comparison of the synaptic transmission of these inputs, with their distinct afferent temporal relays (Fig. 1), will provide insight into the central impact of ADS and its regulation by CFA. Lamina I NK1R+ neurons were preidentified using TMR-SP, which is not expected to alter recorded synaptic activity (Tong and MacDermott, 2006) and has been used previously (Tong and MacDermott, 2006; Torsney and MacDermott, 2006; Torsney, 2011; Dickie and Torsney, 2014; Peirs et al., 2015).

**Figure 2.**
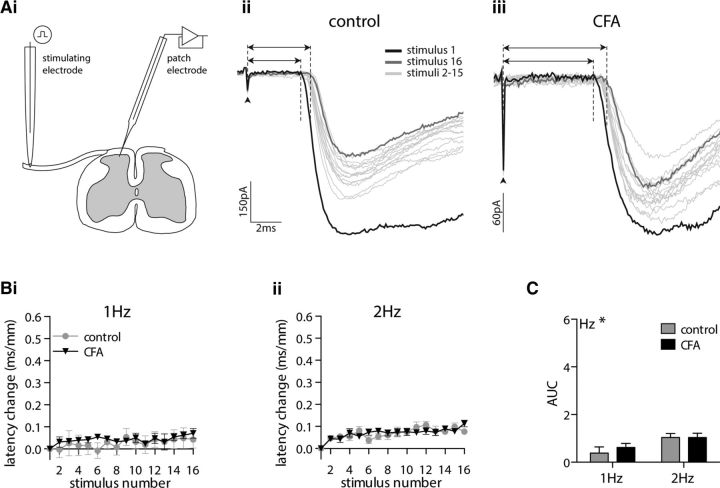
CFA inflammation does not alter limited ADS in monosynaptic Aδ fiber input to lamina I NK1R+ neurons. ***Ai***, eEPSCs were recorded from prelabeled lamina I NK1R+ neurons in spinal cord slices in response to stimulation of attached dorsal roots. ***Aii***, ***Aiii***, Representative monosynaptic Aδ fiber eEPSCs recorded in tissue isolated from control (***Aii***) and CFA-treated (***Aiii***) rats. Each trace comprises 16 traces recorded in response to dorsal root stimulation at 2 Hz. Dashed lines and arrows denote the latency of the first and last trace, measured as the time between the stimulus artifact (denoted by the arrowhead) and the onset of the monosynaptic response. ***Bi***, ***Bii***, Stimulation of monosynaptic Aδ fiber input to lamina I NK1R+ neurons at 1 Hz (***Bi***) or 2 Hz (***Bii***) results in a small degree of ADS. ***C***, AUC analysis of latency change reveals that CFA inflammation does not affect the small frequency-dependent progressive latency change (2-way ANOVA; CFA, *p* = 0.570; frequency, *p = 0.016). 1 Hz: control, *n* = 7; CFA, *n* = 12; 2 Hz: control, *n* = 13; CFA, *n* = 22. Note that scaling in ***B*** and ***C*** is identical to that in Figure 3.

Monosynaptic Aδ fiber eEPSCs were recorded, in spinal cord slices from control and CFA-treated rats (Fig. 2*A*), in response to dorsal root stimulation at frequencies of 1 and 2 Hz. This resulted in a small, frequency-dependent (*p* = 0.016), progressive increase in the eEPSC latency that was unaffected by CFA inflammation (*p* = 0.570; Fig. 2*B*,*C*).

**Figure 3.**
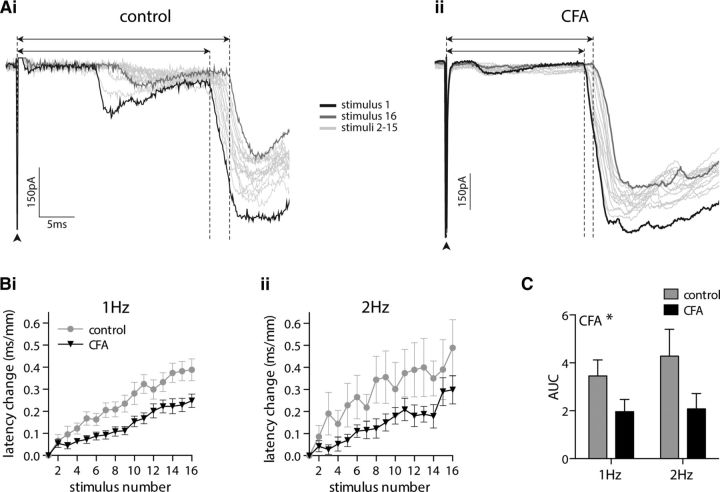
CFA inflammation reduces ADS in monosynaptic C fiber input to lamina I NK1R+ neurons. ***Ai***, ***Aii***, Representative monosynaptic C fiber eEPSCs recorded in tissue isolated from control (***Ai***) and CFA-treated (***Aii***) rats. Each trace comprises 16 traces recorded in response to repetitive dorsal root stimulation at 1 Hz. Dashed lines and arrows denote the latency of the first and last trace, measured as the time between the stimulus artifact (denoted by the arrowhead) and the onset of the monosynaptic response. ***Bi***, ***Bii***, Stimulation of monosynaptic C fiber input to lamina I NK1R+ neurons at 1 Hz (***Bi***) or 2 Hz (***Bii***) results in a progressive increase in response latency. ***C***, AUC analysis of latency change reveals that CFA attenuates ADS, but it is not affected by stimulation frequency (2-way ANOVA; CFA, *p = 0.013; frequency, *p* = 0.521). 1 Hz: control, *n* = 42; CFA, *n* = 64; 2 Hz: control, *n* = 16; CFA, *n* = 36.

In contrast, repetitive stimulation of monosynaptic C fiber input to lamina I NK1R+ neurons resulted in a progressive increase in response latency that was not altered by stimulation frequency (*p* = 0.521) but was markedly reduced by CFA inflammation (*p* = 0.013; Fig. 3) similar to the population C fiber CAP recordings. Notably, the initial conduction velocity of monosynaptic C fiber input to lamina I NK1R+ neurons was not altered by CFA inflammation (*p* = 0.764, Mann–Whitney *U* test; data not shown), as demonstrated previously (Torsney, 2011). CFA inflammation also did not alter the initial peak amplitude of C fiber eEPSCs (*p* = 0.568, Mann–Whitney *U* test; data not shown), as reported previously (Torsney, 2011; Dickie and Torsney, 2014), or the eEPSC amplitude observed during repetitive stimulation (*p* = 0.178, two-way ANOVA; data not shown). In summary, CFA inflammation does not alter the baseline conduction velocity (CV) of monosynaptic C fiber inputs or their eEPSC peak amplitudes in lamina I NK1R+ neurons, but it significantly reduces the progressive delay in synaptic transmission observed between C fibers and lamina I NK1R+ neurons.

### CFA inflammation reduces both the average and range of ADS within the C fiber population

Longer stimulus trains and higher frequencies produce a more pronounced C fiber ADS that is associated with the occurrence of conduction failures (Thalhammer et al., 1994; Nakatsuka et al., 2000; Zhu et al., 2009), the prevalence of which can be altered in pain models (Sun et al., 2012; Wang et al., 2016a), which could additionally impact spinal pain processing. Therefore, to investigate the potential impact of conduction failures, longer stimulus trains and higher frequencies were used.

**Figure 4.**
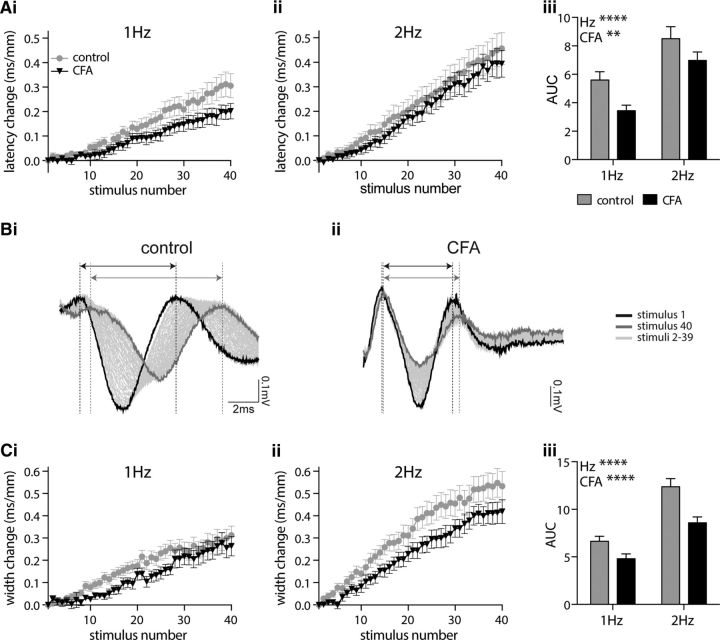
CFA inflammation reduces the average and range of ADS within the C fiber population. ***Ai–Aiii***, C fiber compound action potentials recorded in response to trains of 40 stimuli delivered at 1 Hz (***Ai***) or 2 Hz (***Aii***) results in a progressive slowing of the response latency. AUC analysis of latency change (***Aiii***) reveals that CFA inflammation reduces the frequency-dependent progressive latency change (2-way ANOVA; CFA, **p = 0.005; frequency, ****p < 0.0001). ***Bi***, ***Bii***, Representative C fiber compound action potentials recorded, in response to 2 Hz stimulation, from dorsal roots isolated from control (***Bi***) and CFA-treated (***Bii***) rats, with dashed lines and arrows indicating the width (positive–positive peak) of the first and last responses. ***Ci–Cii***, Trains of 40 stimuli delivered at 1 Hz (***Ci***) or 2 Hz (***Cii***) result in a progressive increase in C fiber compound action potential width. ***Ciii***, AUC analysis of width change reveals that CFA inflammation reduces the frequency-dependent progressive width increase (2-way ANOVA; CFA, ****p < 0.0001; frequency, ****p < 0.0001). Control, *n* = 18; CFA, *n* = 16.

In CAP recordings, stimulating isolated dorsal roots with trains of 40 stimuli delivered at 1 and 2 Hz resulted in a progressive increase in the C fiber response latency, which was frequency dependent (*p* < 0.0001) and significantly reduced by CFA inflammation (*p* = 0.005; Fig. 4*A*). It was not possible to use higher-frequency stimulation in the CAP recordings as the C fiber component of the compound action potential diminishes substantially during repetitive stimulation, presumably because of conduction failures, and as such could only be reliably quantified at 1 and 2 Hz. The change in C fiber response latency reflects the change in average conduction velocity of the population C fiber response. Given that different C fibers display different degrees of ADS (Thalhammer et al., 1994; Serra et al., 1999; Weidner et al., 1999), the change in width of the C fiber response was also measured (Fig. 4*B*), as this will reflect the change in range of conduction velocities present within the population and may, therefore, be a more informative measure of ADS across the entire C fiber population. Stimulation of 1 and 2 Hz resulted in a progressive increase in the C fiber response width, which was also regulated in a frequency- and CFA inflammation-dependent manner (both *p* < 0.0001; Fig. 4*C*). Notably, the initial width of the C fiber response was not significantly different between control and CFA tissue (*p* = 0.27, unpaired *t* test; data not shown).

### CFA inflammation limits the progressive delay in synaptic transmission between C fibers and lamina I NK1R+ neurons without altering synaptic response failures

**Figure 5.**
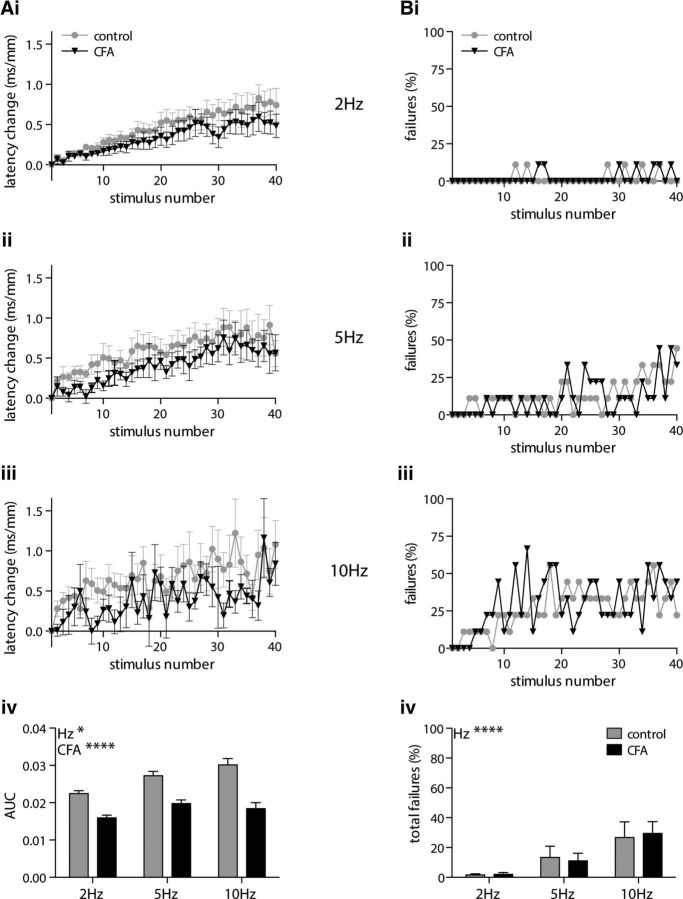
CFA inflammation reduces ADS in monosynaptic C fiber input to lamina I NK1R+ neurons during high-frequency stimulus trains but does not alter synaptic response failures. ***Ai–Aiv***, Repetitive stimulation of monosynaptic C fiber input to lamina I NK1R+ neurons, using trains of 40 stimuli, delivered at 2 Hz (***Ai***), 5 Hz (***Aii***), or 10 Hz (***Aiii***), results (***Aiv***) in a frequency-dependent progressive increase in the eEPSC latency that is significantly reduced by CFA inflammation (2-way ANOVA; CFA, ****p < 0.0001; frequency, *p = 0.039). ***Bi–Biii***, There is a progressive increase in the number of synaptic response failures per stimulus number during stimulation of monosynaptic C fiber input to lamina I NK1R+ neurons at 2 Hz (***Bi***), 5 Hz (***Bii***), or 10 Hz (***Biii***). ***Biv***, The total number of synaptic response failures is frequency dependent but not altered by CFA inflammation (2-way repeated-measures ANOVA; CFA, *p* = 0.971; frequency, ****p < 0.0001). All groups, *n* = 9.

In eEPSC recordings, repetitive stimulation (40 times) of the monosynaptic C fiber input to lamina I NK1R+ neurons at 2, 5, and 10 Hz resulted in a frequency-dependent ADS (*p* = 0.039) that was reduced by CFA inflammation (*p* < 0.0001; Fig. 5*A*). Plotting the percentage of lamina I NK1R+ neurons displaying synaptic response failures per stimulus number reveals a progressive increase in the number of failures (Fig. 5*Bi–Biii*). This progressive increase in synaptic response failures is, as expected, frequency dependent, with the total number of failures per lamina I NK1R+ neuron increasing with stimulation frequency (*p* < 0.0001). However, the total number of synaptic response failures was not affected by CFA inflammation (*p* = 0.971; Fig. 5*Biv*).

### C fiber ADS is regulated in a sex- and inflammation-dependent manner

**Figure 6.**
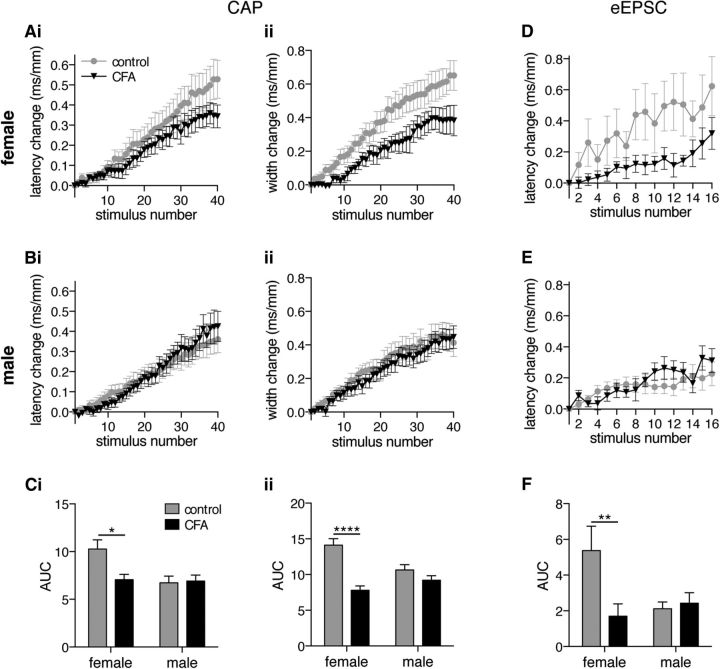
CFA inflammation reduces ADS, within the C fiber population and in the monosynaptic C fiber input to lamina I NK1R+ neurons, in females only. C fiber compound action potentials recorded in response to 2 Hz trains (40 times stimuli) in females (***A***) and males (***B***) results in a progressive slowing of the response latency (***i***) and width ***(ii)***. ***Ci***, ***Cii***, AUC analysis of latency change (***Ci***) and width change (***Cii***) reveals a significant interaction between sex and inflammation (2-way ANOVA; latency change, *p* = 0.037; width change, *p* = 0.003) with CFA inflammation reducing ADS in females only (latency change: females, *p = 0.031; males, *p* = 0.998; width change: females, ****p < 0.0001; males, *p* = 0.525; Tukey's multiple comparisons test). ***D***, ***E***, Monosynaptic eEPSCs in lamina I NK1R+ neurons recorded in response to 2 Hz trains (40 times stimuli) results in a progressive slowing of the response latency in females (***D***) and males (***E***). ***F***, AUC analysis of latency change reveals a significant interaction between sex and inflammation (2-way ANOVA, *p* = 0.034) with CFA inflammation reducing ADS in females only (female, **p = 0.0057; male, *p* = 0.996; Tukey's multiple comparisons test). CAP: female control, *n* = 9; female CFA, *n* = 7; male control, *n* = 9; male CFA, *n* = 9. eEPSC: female control, *n* = 10; female CFA, *n* = 17; male control, *n* = 6; male CFA, *n* = 16.

To determine whether there is a sex difference in the ADS phenomenon CAP (Fig. 6*A–C*) and eEPSC (Fig. 6*D–F*), datasets were analyzed for females and males separately. In CAP recordings from isolated dorsal roots, a progressive increase in the latency and width of the population C fiber response was observed in both females (Fig. 6*Ai*,*Aii*) and males (Fig. 6*Bi*,*Bii*). AUC analysis reveals a significant interaction between sex and CFA inflammation for both latency change (Fig. 6*Ci*; *p* = 0.037) and width change (Fig. 6*Cii*; *p* = 0.003). ADS is more pronounced in control females, and CFA inflammation reduces ADS, in females only, to a level that is now comparable with males for both latency change (CFA: female, *p* = 0.031; male, *p* = 0.998) and width change (CFA: female, *p* < 0.0001; male, *p* = 0.525) measures. Initial CV and CAP width were not altered in a sex- or CFA inflammation-dependent manner (CV: sex, *p* = 0.853; CFA, *p* = 0.120; width: sex, *p* = 0.482; CFA, *p* = 0.307; two-way ANOVA; data not shown).

Similarly, patch-clamp recordings from lamina I NK1R+ neurons show a progressive increase in the latency of monosynaptic C fiber-evoked eEPSCs in both females (Fig. 6*D*) and males (Fig. 6*E*). Likewise, AUC analysis reveals a significant interaction between sex and CFA inflammation (Fig. 6*F*; *p* = 0.034). ADS is more pronounced in control females, and CFA inflammation reduces ADS, in females only, to a level that is now comparable with males (CFA: female, *p* = 0.0057; male, *p* = 0.996). However, baseline CV of monosynaptic C fiber inputs was not altered in a sex- or CFA inflammation-dependent manner (sex, *p* = 0.873; CFA, *p* = 0.923; data not shown).

### Limiting ADS facilitates spinal summation

**Figure 7.**
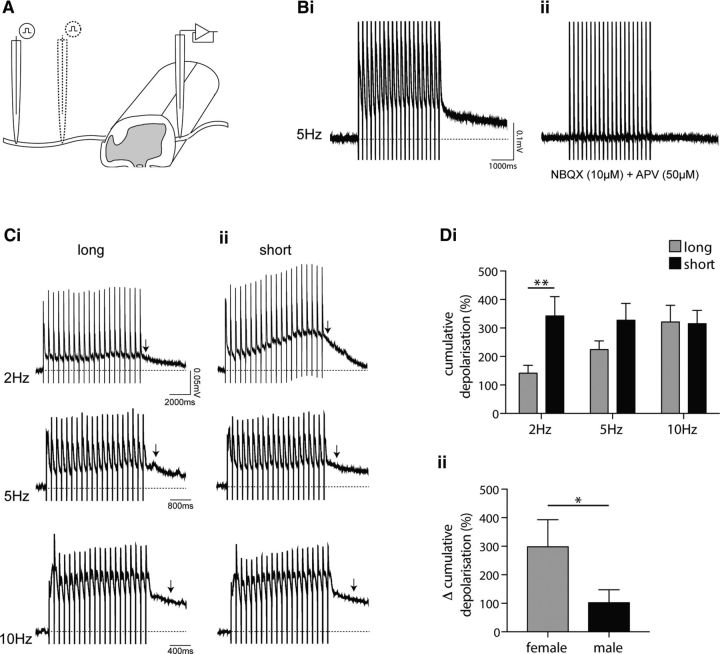
Cumulative dorsal root-evoked ventral root potentials are regulated in a frequency- and length-dependent manner. ***A***, Ventral root potentials were recorded from a hemisected spinal cord preparation in response to long length and short length dorsal root stimulation (20 times) to increase and decrease the spinal impact of ADS, respectively, using suction electrodes. ***Bi***, ***Bii***, Representative cumulative dorsal root-evoked (5 Hz) ventral root potential (***Bi***) that is abolished in the presence of NBQX (10 μm) and APV (50 μm; ***Bii***). Stimulus artifact is visible as vertical lines in all traces. ***Ci***, ***Cii***, Representative cumulative dorsal root-evoked ventral root potentials induced by 2, 5, and 10 Hz stimulation over long (***Ci***) and short (***Cii***) dorsal root lengths. Arrows denote the 500 ms time point, after the last stimulus artifact, when amplitude is measured. ***Di***, Repeated-measures ANOVA of normalized cumulative ventral root potentials reveals both frequency (*p* = 0.039)- and length (*p* = 0.003)-dependent effects that significantly interact (*p* = 0.022) with Sidak's multiple comparisons test revealing a significant impact of length at 2 Hz only (**p < 0.01; *n* = 10). ***Dii***, Difference in percentage cumulative depolarization between long and short stimulation lengths at 2 Hz, is significantly greater in females (1-tailed Mann–Whitney *U* test, *p = 0.028; *n* = 5 both groups).

Our observations demonstrate that ADS results in a progressive delay in the synaptic transmission of C fiber input to individual spinal neurons. Moreover, given that repetitive C fiber stimulation results in a progressive increase in C fiber CAP width, which reflects the range of CVs within the population, ADS also likely reduces the temporal coincidence of population C fiber input at a spinal network level. Together, these findings suggest that ADS limits temporal summation of C fiber-evoked synaptic activity at a spinal level. Therefore, reducing C fiber ADS should enhance spinal summation. To test this hypothesis, we took advantage of the length dependency of ADS (Schmelz et al., 1995; Zhu et al., 2009) and assessed the impact of long (increased ADS) versus short (decreased ADS) root stimulation lengths on spinal summation by recording cumulative dorsal root-evoked ventral root potentials in a hemisected spinal cord preparation (Fig. 7*A*). Using this preparation, we are essentially able to assess the impact of ADS on the circuitry underlying the nociceptive flexion withdrawal reflex.

Repetitive stimulation of dorsal roots evoked a cumulative ventral root potential that is blocked, as expected (Thompson et al., 1992), in the presence of the AMPA receptor antagonist NBQX (10 μm) and NMDA receptor antagonist APV (50 μm; Fig. 7*B*). This spinal summation is frequency dependent (Fig. 7*Ci*), as previously reported (Thompson et al., 1992). Interestingly, reducing the stimulation length strongly facilitated the cumulative ventral root potential at 2 Hz stimulation rates (Fig. 7*Cii*). To quantify the degree of summation, the amplitude of the cumulative ventral root potential was measured 500 ms after the last stimulus artifact and normalized to the amplitude measured at 500 ms after a single stimulus at long or short root stimulation length to account for any potential difference in response amplitude between stimulation lengths. Repeated-measures ANOVA revealed a significant interaction (*p* = 0.022) between frequency (*p* = 0.039)- and length (*p* = 0.003)-dependent effects, and post-tests revealed a significant difference between long and short lengths at 2 Hz stimulation rates only (Fig. 7*Di*; 2 Hz, *p* < 0.01). Given the more pronounced C fiber ADS in females, we predicted a greater change in facilitation of spinal summation in females versus males. Indeed, there is a greater difference in the percentage cumulative depolarization between long and short stimulation length in females at the 2 Hz stimulation rate (Fig. 7*Dii*; *p* = 0.028).

To estimate the degree of change in ADS resulting from the average ∼50% reduction in root stimulation length in the dorsal root–ventral root potential (DR-VRP) recordings and thus the likely relevance of the findings with respect to our sex/inflammation-dependent changes in ADS, we revisited the two times length stimulation CAP recordings displayed in Figure 1*D*. In the CAP recordings, a comparable reduction in stimulation length (∼60%) reduced ADS by ∼20% as measured using AUC analysis of latency change (unpaired *t* test, *p* = 0.007; data not shown). Therefore, in these DR-VRP recordings, the short stimulation length likely underestimates the average ∼30% reduction in ADS compared with the control female grouping (also assessed using AUC analysis of latency change; Fig. 6*Ci*), underscoring the importance of these findings.

### Females display elevated noxious thermal thresholds and enhanced inflammatory thermal hyperalgesia

The demonstration that females display more pronounced ADS that is reduced by CFA inflammation to a level that is comparable with males (control and CFA) along with the observation that limiting ADS facilitates cumulative dorsal root-evoked ventral root potentials, the following predictions can be made regarding the nociceptive flexion withdrawal reflex: (1) control females should have higher pain thresholds than control males; (2) if other underlying inflammatory pain mechanisms are not sex dependent, then CFA inflammation-reduced pain thresholds should be similar between males and females; and (3) because these predictions imply a greater reduction in pain threshold in females versus males, females should display enhanced inflammatory hypersensitivity.

**Figure 8.**
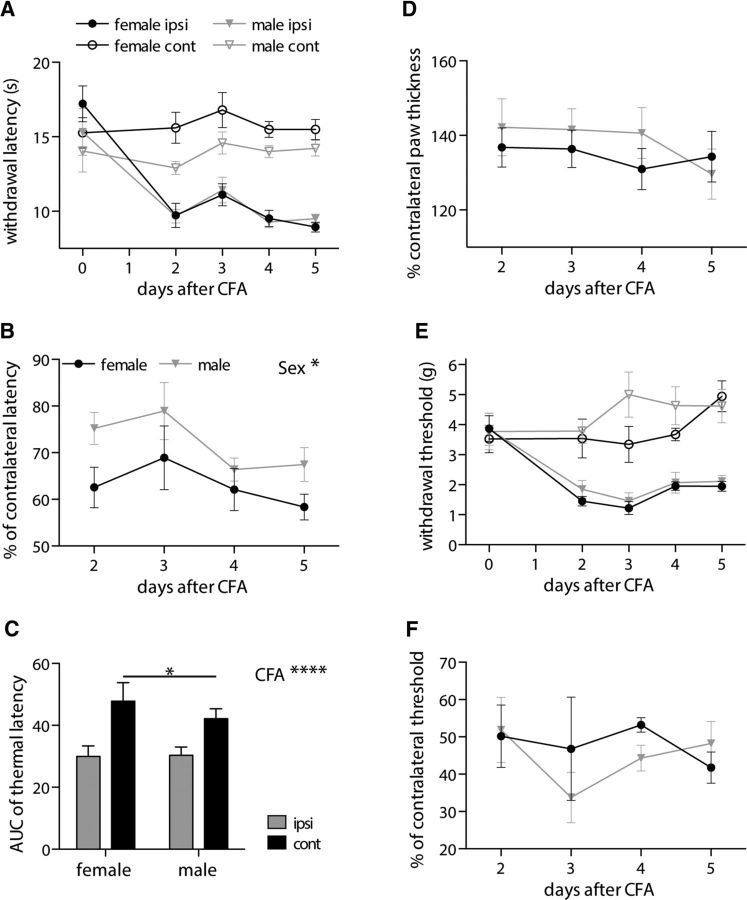
Behavioral analysis of the nociceptive flexion withdrawal reflex demonstrates elevated thermal pain thresholds and enhanced inflammatory thermal hyperalgesia in females. ***A***, Ipsilateral (ipsi) and contralateral (cont) hindpaw withdrawal latencies to noxious radiant heat stimuli (Hargreaves) were measured in female and male rats before and 2–5 d after CFA intraplantar injection. ***B***, Ipsilateral withdrawal latency values expressed as a percentage of contralateral values reveal a sex difference in thermal hyperalgesia (repeated-measures ANOVA, *p = 0.032). ***C***, AUC analysis (2–5 d after CFA, ****p < 0.0001) shows a significant sex difference in contralateral hindpaw withdrawal latency with higher values in females (2-way ANOVA, followed by Sidak's multiple comparisons test, *p = 0.022). ***D***, Ipsilateral hindpaw thickness is expressed as a percentage of contralateral values and demonstrates that CFA-induced paw swelling is not sex dependent (repeated-measures ANOVA, *p* = 0.513). ***E***, Ipsilateral and contralateral hindpaw mechanical thresholds (von Frey) were also measured in the same female and male rats before and 2–5 d after CFA intraplantar injection. ***F***, Ipsilateral mechanical thresholds are expressed as a percentage of contralateral values and demonstrate that mechanical inflammatory hypersensitivity is not sex dependent (repeated-measures ANOVA, *p* = 0.548). All graphs: female, *n* = 9; male, *n* = 7.

Figure 8*A–C* displays behavioral data obtained in response to noxious radiant heat stimuli in the CFA inflammation model that supports these predictions. Females show increased inflammatory thermal hypersensitivity (Fig. 8*B*; *p* = 0.032) that, importantly, does not appear to be attributable to an increased inflammatory response because CFA-induced paw swelling is not significantly different between sexes (Fig. 8*D*; *p* = 0.513). It instead reflects a larger reduction in thermal pain thresholds from an elevated baseline level as can be observed by comparing contralateral male and female withdrawal latencies (Fig. 8*A*). Notably, AUC analysis of thermal withdrawal latency values (2–5 d after CFA) reveals a significant interaction (*p* = 0.044) between sex and hindpaw (ipsilateral/contralateral) with post-tests revealing significantly higher control/contralateral hindpaw values in females (*p* = 0.022). The mechanical threshold of the flexion withdrawal reflex was assessed using von Frey monofilaments (Fig. 8*E*). AUC analysis reveals no significant interaction between sex and hindpaw (repeated-measures ANOVA, *p* = 0.180; data not shown). Furthermore, inflammatory mechanical hypersensitivity was not altered in a sex-dependent manner (Fig. 8*F*; *p* = 0.548).

## Discussion

We found that C fiber ADS is more pronounced in females than males and is reduced by CFA inflammation in females only. This alters the timing of synaptic transmission of monosynaptic C fiber input to lamina I NK1R+ neurons. Experimental manipulation of ADS demonstrates it can influence spinal summation consistent with observed sex differences in noxious thermal thresholds. We propose that ADS regulates nociceptive drive to central pain circuits.

### Underlying mechanisms of ADS

The underlying mechanism of ADS is not fully understood. It was initially proposed to result from Na^+^–K^+^–ATPase-dependent membrane hyperpolarization (Rang and Ritchie, 1968; Bostock and Grafe, 1985), but Na^+^-K^+^-ATPase blockade increased rather than reduced ADS (De Col et al., 2008). Pharmacological studies then suggested ADS reflects increased numbers of Nav channels entering a slow-inactivated state (De Col et al., 2008; Obreja et al., 2012). However, modeling suggested that although Nav channel slow inactivation contributes, ADS is most readily associated with an increase in intra-axonal Na^+^ concentration (Tigerholm et al., 2014). Investigation of the differences between mechano-insensitive C fibers that display the greatest ADS versus mechano-sensitive C fibers that display minimal ADS (Weidner et al., 1999; Obreja et al., 2010) revealed that pronounced ADS was associated with more Nav1.8, less Nav1.7, more delayed rectifier potassium channel, and less Na^+^/K^+^ ATPase, a profile consistent with enhanced accumulation of intracellular Na^+^ (Petersson et al., 2014). Notably, given the observed sex/inflammation-dependent ADS, these molecules are regulated by peripheral inflammation (Coggeshall et al., 2004; Gould et al., 2004; Zhang and Nicol, 2004; Liang et al., 2013; Rahman and Dickenson, 2013; Waxman and Zamponi, 2014; Wang et al., 2015), but, despite being key pharmaceutical targets, sex differences in their regulation in C fiber nociceptors have not been explored.

In addition to the aforementioned molecules implicated in ADS, there are many other ion channels/receptors whose expression, location, and/or function can be altered by inflammation (Gold and Gebhart, 2010) and may contribute to altered ADS. Furthermore, previous studies have reported CFA inflammation induced changes in C fiber nociceptor excitability, including increased CV combined with reduced electrical thresholds in guinea pig (Djouhri and Lawson, 2001) not replicated in this or other rat studies (Baba et al., 1999; Nakatsuka et al., 2000; Torsney, 2011), altered action potential shape (Djouhri and Lawson, 1999; Zhang et al., 2012), and spontaneous firing (Djouhri et al., 2006; Xiao and Bennett, 2007; Matson et al., 2015). Moreover, estrogen exacerbates inflammation-increased excitability of temporomandibular joint afferents (Flake et al., 2005), and there are sex differences in inflammatory sensitization of dural afferents (Scheff and Gold, 2011).

### Evidence for altered ADS

Consistent with our observed inflammation-altered ADS, the inflammatory mediator NGF reduces ADS in the mechano-insensitive C fibers that normally display pronounced ADS (Obreja et al., 2011) and is associated with less Nav1.8, more Nav1.7, less delayed rectifier potassium channel, and more Na^+^/K^+^ ATPase (Petersson et al., 2014). Furthermore, there is reduced ADS in a diabetic neuropathic pain model (Wang et al., 2016b). In contrast, there is enhanced ADS in the spinal nerve ligation model of neuropathic pain (Shim et al., 2007), but these findings need to be considered within a partially denervated pain circuitry. Microneurography studies have also demonstrated altered ADS in chronic pain patients (Ørstavik et al., 2003, 2006; Kleggetveit et al., 2012; Serra et al., 2014), interestingly, including patients with mutations in Nav1.7 (Namer et al., 2015) and Nav1.8 (Kist et al., 2016).

### Physiological relevance of ADS

C fiber ADS is physiologically relevant because it occurs not only after electrical stimulation but also in response to natural stimulation of the skin (Thalhammer et al., 1994). Electrical stimulation-induced ADS is useful as it can provide a readout of nociceptor excitability given the key roles of molecules implicated in ADS, such as Nav and HCN channels, in action potential generation and regulation of initial firing frequency in nociceptor terminals. It is not surprising, therefore, that altered ADS profiles are associated with changes in C fiber thresholds (Obreja et al., 2011), that ADS correlates with spontaneous firing (Kleggetveit et al., 2012), and that ADS is accompanied by a parallel increase in C fiber mechanical threshold (De Col et al., 2012).

Moreover, ADS is proposed to provide a memory trace of previous activity levels that can influence responses to subsequent inputs. Specifically, low-level firing comparable with spontaneous firing rates in inflammatory pain induces ADS that dynamically influences the response to higher-frequency inputs such that they display reduced ADS or even speeding (Weidner et al., 2002). Importantly, in our data, inflammation-induced spontaneous C fiber firing will likely be absent, attributable to the lack of peripheral inflamed tissue, in our *ex vivo* preparations. Therefore, our observations may well underestimate the degree of inflammation-reduced ADS as our recorded data likely only reflect the inflammation-altered expression levels of ion channels involved in ADS and not the dynamic regulation of ADS by ongoing C fiber activity.

Here we demonstrate that, in addition to ADS providing a nociceptor excitability readout and a dynamic memory, the progressive slowing per se significantly influences the relay of nociceptive signals such that the firing frequency initiated in the periphery is not faithfully transmitted to the spinal cord. In individual spinal neurons, there is a progressive delay in synaptic transmission of C fiber inputs, and we also observe a progressive reduction in temporal coincidence of population C fiber input that we predicted should alter summation at a spinal network level. Decreasing stimulation length in DR-VRP recordings demonstrated that reducing ADS did indeed facilitate spinal summation. Interestingly, facilitation was only significant at the 2 Hz stimulation rate and, strikingly, increased summation to a level comparable with that observed at 10 Hz. The lack of effect at higher frequencies probably reflects the increased incidence of synaptic failures with increasing stimulation frequency (Fig. 5*B*), likely resulting from frequency-dependent C fiber conduction failure (Nakatsuka et al., 2000; Zhu et al., 2009; Sun et al., 2012; Wang et al., 2016a) that will limit the maximal extent of spinal summation. Notably, spontaneous firing rates resulting from tissue inflammation are typically <1 Hz (Djouhri et al., 2006; Xiao and Bennett, 2007; Matson et al., 2015), whereas noxious stimuli evoke firing in the 1–10 Hz range (Lynn and Carpenter, 1982; Leem et al., 1993). We therefore propose that reducing ADS thereby promotes temporal coincidence of nociceptive input, as a novel mechanism of hyperalgesia whereby lower-intensity noxious inputs can be amplified but high-intensity inputs are not further amplified because of transmission failures, which may reflect an intrinsic self-inhibition mechanism to limit overdrive of the nociceptive pathways. Notably, ADS was altered in the monosynaptic C fiber input to lamina I NK1R+ likely projection neurons that display tightly controlled spike timing-dependent plasticity (Li and Baccei, 2016). Therefore, these changes in ADS may influence the involvement of these output neurons in synaptic plasticity (Ikeda et al., 2003, 2006), spinal supraspinal loop activity (Suzuki et al., 2002), and chronic pain (Mantyh et al., 1997; Nichols et al., 1999). Finally, promotion of closely timed nociceptive inputs appears to be a feature after injury with the recent demonstration of novel DRG neuronal coactivation after CFA inflammation that contributes to mechanical hyperalgesia (Kim et al., 2016).

The pronounced ADS in control females that was reduced by CFA to male levels, along with the observation that limiting ADS facilitates spinal summation, was consistent with behavioral observations of elevated noxious thermal latencies and enhanced inflammatory thermal hyperalgesia in females. Whereas the enhanced hypersensitivity is consistent with sex differences in human studies, the elevated noxious thermal latencies are not (Mogil, 2012; Bartley and Fillingim, 2013). However, we studied spinal reflex behaviors in which the afferent input is a major component of the underlying neural circuitry, whereas human subjective pain scoring additionally involves higher-level cognitive processing. If differing degrees of ADS contributes to sex differences in pain thresholds, it is not surprising that this is specific for thermal versus mechanical sensitivity because the inflammatory mediator NGF can reduce ADS in mechano-insensitive C fibers that can respond to heat (Schmidt et al., 1995; Schmelz et al., 1997) without unmasking of mechanical sensitivity (Obreja et al., 2011). However, the lack of sex differences in mechanical sensitivity may reflect the use of von Frey hairs to identify mechanical thresholds rather than a noxious mechanical stimulus. Alternatively, it may be that different peripheral mechanisms, DRG neuronal coupling and reduced ADS, are used to augment noxious mechanical and thermal inputs, respectively. It will be important to determine the extent to which ADS regulates the processing of noxious stimuli *in vivo* and also identify the mechanisms underlying sex-dependent ADS and whether these are controlled by sex hormones or genetic factors.

It has been long established that the intensity of a sensation is encoded by afferent firing frequency. Here we propose that the firing frequency initiated in the periphery is diminished en route to the CNS by ADS in nociceptive C fibers and that inflammation/sex-dependent regulation of ADS can thereby peripherally modulate spinal processing and pain sensation.
